# Do Childcare Teachers Evaluate Children’s Weight Status More Accurately Than Parents? A Brief Report

**DOI:** 10.1177/08901171231178272

**Published:** 2023-05-21

**Authors:** Ana I. Gomes, Rosa Lemos, Milica Miočević, Ana I. Pereira, Luísa Barros

**Affiliations:** 1 CICPSI, 37810 Faculdade de Psicologia, Universidade de Lisboa, Lisboa, Portugal; 2 37810 Faculdade de Psicologia, Universidade de Lisboa, Lisboa, Portugal; 3 Department of Psychology, 5620 McGill University, Montreal, QC, Canada

**Keywords:** perception of the child’s weight, childcare teachers, parents, children, kindergarten

## Abstract

**Purpose:**

Parents’ underestimation of young children’s weight can reduce their engagement and readiness to implement changes in children’s diet and physical activity. Childcare teachers can support parents’ identification of children at risk for being overweight only if they can accurately do this themselves.

**Design:**

Quantitative, cross-sectional study.

**Setting:**

Fifteen kindergartens near Lisbon, Portugal.

**Subjects:**

319 parents, 32 teachers (47.5% and 100% response rate, respectively), and 319 children.

**Measures:**

Caregivers classified the children’s weight, considering their height and age as underweight, healthy weight, or overweight; children’s body mass index (BMI) status for age and sex was assessed.

**Analysis:**

Differences in caregivers’ accuracy of children’s weight perception were assessed. Multilevel multivariate logistic regression models were used to analyze the predictors of the accuracy of teachers’ and parents’ weight perception as a binary outcome.

**Results:**

The proportion of children with overweight correctly assessed differed significantly (*P* = 0.004) between teachers (31.1%) and parents (17.5%). The child’s BMI percentile was the only significant positive predictor for both caregivers’ weight perception accuracy (*P* < 0.001 and *P* = 0.004, for parents and teachers, respectively), holding the child’s age and sex constant.

**Conclusion:**

Although childcare teachers were better raters than parents when evaluating children’s weight status, the percentage of children with overweight that childcare teachers misclassified was still relatively high.

## Purpose

Parental perception of their child’s weight has been widely studied, showing two overall conclusions. Parents tend to underestimate their child’s weight,^1^ and this bias can influence their readiness to implement changes in children’s diet, physical activity,^2,3^ and feeding practices.^4^ Therefore, parents may need help identifying their child’s weight status and recognizing when the child has overweight or obesity. Childcare teachers can play a relevant role in this process as they collaborate closely with families, supporting parents on children’s development, education, and health issues.^5^ However, to properly advise parents about their children’s weight issues, teachers must be able to identify children who might be at increased risk of non-healthy weight. Although we could not find any study about childcare teachers’ perception of children’s weight, earlier contributions showed that teachers were generally skeptical about the definition of overweight in young children, were unsure about how to address this issue,^6^ and had difficulty accepting that a child has overweight even when a healthcare professional identified this condition.^7^ This study aimed to determine how accurately childcare teachers perceive preschool children’s weight status compared with parents and how caregivers’ accuracy perception varied according to children’s BMI, sex, and age.

## Methods

### Design

We invited parents of children who attended 15 public and state-funded kindergartens near Lisbon, Portugal, to participate through a leaflet explaining the study aims and the conditions to participate and a written informed consent form. Parents who returned the signed informed consent form received a questionnaire to be filled out and sent back to the school. A similar procedure was performed with the childcare teachers of these children.

### Sample

Of the 743 parents invited, 353 agreed to participate in the study (47.5% response rate); 349 responses were considered eligible. We measured 346 children whose parents completed the questionnaire. All children gave their oral assent before measurement. All children’s teachers agreed to collaborate (100% response rate); 321 responses were eligible. In the end, we collected complete data from 319 triads (i.e., 319 children, their parents, and 32 teachers).

Childcare teachers were all female and had a first degree (4 years) or a postgraduate degree in early childhood education; 78.2% were aged 35 to 54. Most parents (92.2%) were mothers aged 25 to 44 (92.5%) and had completed a higher education degree (46.4%). The children’s mean age was 4.45 years (SD = 0.845).

### Measures

We collected sociodemographic data regarding parents, teachers, and children. Both caregivers were asked about their perception of the child’s current weight considering their age and sex (i.e., underweight, healthy weight, overweight).^8^ A trained nutritionist weighed and measured children in light clothing and barefoot with a SECA model 220 column scale with a stadiometer. To calculate BMI-for-age-and-sex percentiles and status, we applied the Centers for Disease Control and Prevention’s standards (the norms adopted in Portugal) and classified children into three groups (to better correspond to the response options available in the parents’ weight status perception question): below the 5^th^ BMI percentile as underweight, over the 85^th^ BMI percentile as overweight and the remaining as a healthy weight.

### Analysis

We used descriptive analysis to characterize the samples regarding sociodemographic data, children’s BMI-for-age-and-sex status, and caregivers’ perceptions of the child’s weight. To assess the differences in the proportions of caregivers’ ratings regarding a child’s weight, we recoded data into two categories (accurate vs inaccurate perception of weight). McNemar’s test was used for children with healthy weight and overweight with a Bonferroni adjustment (*P* < 0.025). The data for children with underweight were not analyzed due to insufficient sample size. These statistical analyses were performed with SPSS for Windows, version 26. The multivariate logistic regression model with caregivers’ accuracy when evaluating children’s weight as binary outcomes (coded so 1 = inaccurate and 0 = accurate perception), predicted by the child’s age, sex, and BMI percentile, was estimated using the R package lavaan.^9^ The prediction analysis was run for parents and teachers separately, being the teacher’s ID the clustering variable for both multilevel models. The function glmer () from the R package lme4^10^ was used for the separate analyses with a logit link.

## Results

Regarding the BMI-for-age-and-sex status, 65.5% of children had a healthy weight, 32.3% had overweight (of which 41.8% had obesity), and 2.2% had underweight. Approximately 2/3 of children were accurately rated by their teachers (67.4%) and parents (65.2%). Correct assessments were mainly of children with a healthy weight (83.3% and 89.4% for teachers and parents, respectively). The incorrect estimates were mostly underestimations of the child’s weight (92.3% of the childcare providers and 97.3% of the parents’ incorrect evaluations) and more frequent for children who were overweight (68.3% and 76.6% for teachers and parents, respectively). The proportion of children with overweight and accurately assessed differed significantly (*P* = 0.004) between teachers (31.1%) and parents (17.5%); this difference was not found for children with a healthy weight (*P* = 0.265).

The multilevel multivariate logistic regression analysis, run separately for parents and teachers, showed that only the child’s BMI percentile was a significant predictor of accuracy when evaluating the child’s weight status in both proposed models. Neither the child’s age nor sex were significant predictors of the caregiver’s accuracy when predicting the child’s weight. Regarding parents, for every unit increase in the child’s BMI percentile, the logit of incorrectly predicting the child’s weight increases by 0.03 (*P* < 0.001), holding the child’s age and sex constant. For teachers, the logit of incorrectly predicting the child’s weight increases by 0.01 (*P* = .004) for every unit increase in percentile BMI child, also holding the same variables constant.

## Discussion

### Summary

Both caregivers incorrectly assessed about one-third of the children. One reason for this misperception can be the children’s young age, as shown before with parents.^8^ The bias was mainly an underestimation of weight and occurred more frequently with children with a non-healthy weight status.^1,6^ Consistently, the only significant predictor of accurate weight status perception was the child’s BMI percentile for both caregivers. Also, teachers were significantly better than parents when evaluating children with overweight. Parents can have more reluctance to acknowledge their child’s weight problem, or may rely on different criteria when appraising their child’s weight.^11^ Also, teachers interact with many more preschool children, which might improve their ability to assess the child’s weight status accurately.

Nevertheless, the relatively high percentage of children with overweight misclassified by teachers is concerning. Teachers who do not timely recognize these signs may fail to implement actions to promote physical activity, use responsive feeding strategies during mealtimes (e.g., helping children identify and respond appropriately to feelings of hunger and fullness instead of pressuring them to eat), and share their concerns with parents. Children eat several meals at school, and the educators’ feeding practices and modeling can influence children’s eating behaviors and diet.^12^ Teachers need to proceed sensitively not to increase obesity-related stigma in young children; however, supporting parents in recognizing their child’s weight status is critical for preventing obesity and other chronic health conditions from the first years of life. Although childcare teachers are not directly responsible for the children’s healthcare, they have a role in supporting families in providing the best care for their children.^5,13^ They are often the first to advise parents on development and health issues.^14^

### Limitations

We used a convenience, non-representative sample. BMI is a conventional anthropometric measure to assess nutritional status in research and a clinical context; however, it is not the only way to evaluate children’s weight development. Although we used the most common measure of parental perception of the child’s weight, relying on a sole question can limit the options for more complex statistical analysis. We only considered children’s variables as predictors of the accuracy of the weight status perception, thus, not considering the influence of caregivers’ dimensions on their child’s weight evaluation.

### Significance

Considering the primary role that childcare teachers play in children’s healthy development and the opportunities for them to advise parents on issues regarding eating and physical activity, they might need more specific training to better evaluate situations where the child’s weight might suggest a health risk. Teachers who are more attentive and prepared to identify issues in children’s weight accurately can better advise parents about children’s health and feeding and support them in implementing timely preventive efforts.

**“So What?”**

**What Is Already Known on This Topic?**

Parents tend to underestimate their child’s weight status. Although childcare teachers play a critical role in supporting parents regarding children’s health promotion, some studies suggest that they also have difficulty recognizing overweight in children, thus hindering their role in helping parent’s recognition of overweight in their children.

**What Does This Article Add?**

The study explores whether childcare teachers are more accurate than parents when evaluating children’s weight and which child characteristics are associated with more erroneous perceptions. Teachers were more accurate than parents in their perception of children’s overweight, though underestimations were still high. Child’s BMI percentile was a significant predictor for both caregivers’ accuracy.

**What Are the Implications for Health Promotion Practice or Research?**

Teachers might benefit from training to improve the timely identification of overweight risk in young children, enabling them to better guide parents on their child’s feeding and physical activity habits and implement prevention efforts early in kindergartens.
